# Evaluation of phototoxicity induced by the anticancer drug rucaparib

**DOI:** 10.1038/s41598-022-07319-9

**Published:** 2022-03-02

**Authors:** Alejandro Mateos-Pujante, María Consuelo Jiménez, Inmaculada Andreu

**Affiliations:** 1 Departamento de Química, Universitat Politècnica de València, Camino de Vera s/n, 46022 Valencia, Spain; 2 Unidad Mixta de Investigación Universitat Politècnica de València-Instituto de Investigación Sanitaria (IIS), La Fe, Hospital Universitari i Politècnic La Fe, Avenida de Fernando Abril Martorell 106, 46026 Valencia, Spain

**Keywords:** Biochemistry, Chemical biology

## Abstract

Rucaparib (RCP) is a potent selective inhibitor of both PARP-1 and PARP-2 enzymes that induces synthetic lethality in cancer cells. It is used for the treatment of breast and ovarian tumors harboring deleterious germline or somatic cancer susceptibility genes mutations. Although RCP has an indole chromophore in its structure, it displays a bathochromic shift of the absorption band towards the UVA region of sunlight, thus extending the active fraction of solar light able to produce photosensitivity reactions. In this context, it is highly interesting to study the photo(geno)toxicity disorders associated with this drug, bearing in mind that, for dermatologists it is crucial to understand the toxicity mechanism to improve clinical management. In the present work, RCP has shown to be potentially phototoxic, as observed in the neutral red uptake phototoxicity test. Moreover, this significant phototoxicity is attributed to both proteins and genomic DNA, as revealed in the protein photooxidation and comet assays. The results obtained are highly relevant concerning RCP photosafety and become clinically important in the context of identification of the cutaneous adverse events that can be associated with the targeted therapies. Interestingly, this is the first example of a PARP inhibitor able to induce photosensitized damage to biomolecules.

## Introduction

Cancer is one of the most prevalent diseases and the second leading cause of death worldwide. Notably, among the treatments for this pathology, cancer chemotherapy has been the major pharmacological advance in the last few decades. However, the drugs used for this purpose have a narrow therapeutic index, and often the response produced is just palliative. In contrast, the more recently introduced targeted therapy inhibits a specific molecular goal, usually a protein with a critical role in tumor growth or progression, and therefore it has more limited nonspecific toxicities.

In this context, Poly(ADP-ribose) polymerase (PARP) inhibitors have emerged as promising antitumoral targeted therapies^1,2^.

The poly(ADP-ribose) polymerases (PARPs) are a large family of multifunctional enzymes that catalyze the transfer of ADP-ribose to target proteins. They are involved in cellular processes such as modulation of chromatin structure, transcription, replication, recombination, and DNA repair^3^. The most abundant PARPs, PARP-1 and PARP-2, play an essential role in DNA single-strand break repair (SSB) via the base excision repair (BER) pathway^4^. An efficient SSB repair is essential for cell survival. In this sense, PARP inhibition results in the accumulation of unrepaired SSB, leading to collapsed replication forks and DNA double-strand breaks (DSB) that are toxic to cells, and the homologous recombination repair (HRR) pathway, in which BRCA1 and BRCA2 genes are key elements, is essential to repair such lesion during cell replication^5^. Thus, inhibition of PARP enzymes is a potentially lethal therapeutic strategy, consisting of provoking chromosomal uncertainty, cell-cycle arrest, and subsequent apoptosis, which seems to be attributable to the persistence of DNA lesions that are normally repaired by homologous recombination^4,6^.

Rucaparib (RCP) is a potent selective inhibitor of both PARP-1 and PARP-2, which induces synthetic lethality in cancer cells that are not able to repair DNA damage by the HRR pathway. It is especially used, for the treatment of breast and ovarian tumors harboring deleterious germline or somatic cancer susceptibility genes (BRCA) mutations^7–11^. Recently, rucaparib has been approved by the Food and Drug Administration (FDA) for the treatment of patients with deleterious BRCA mutation associated with metastatic castrate-resistant prostate cancer (mCRPC)^12^.

Generally, RCP is well tolerated by patients but some adverse events can occur, including fatigue, dizziness, gastrointestinal disorders, thrombocytopenia, neutropenia, itching and skin sensitivity to sunlight^13^. With this background, the present work aims to investigate photodamage to biochemical targets in living cells, using a methodology previously developed in our group to study photosensitivity reactions induced by drugs and metabolites^14–18^.

**Figure 1:**
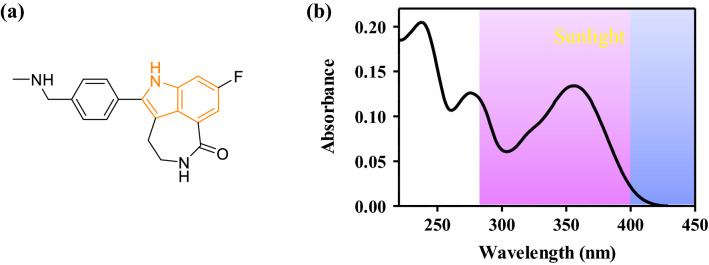
(**a**) Chemical structure of rucaparib (RCP). (**b**) Absorption spectrum of RCP 20 μM in PBS. The spectrum was drawn using GraphPad Prism v5.03.

From a photobiological point of view, although RCP has an indole chromophore in its structure, it displays a bathochromic shift of the absorption band towards the region of sunlight, (UVA) being able to induce photosensitivity (Fig. 1). Therefore, it appears necessary to investigate the RCP photosensitivity disorders (phototoxicity and photogenotoxicity), contributing to a better understanding of the photosensitivity phenomena. These findings will help dermatologists to improve clinical management in oncologic patients.

## Methods

### General

All solvents were commercially available (HPLC grade) and were used without further purification. Rucaparib (RCP, CAS no. 283173-50-2) was purchased from Cymit Química S.L. (Barcelona, Spain). Sodium dodecyl sulphate (SDS, CAS no. 151-21-3) chlorpromazine hydrochloride (CPZ, CAS no. 69-09-0) and 5-hydroxydiclofenac (5-OH DCF, CAS no. 69002-84-2) were purchased from Sigma-Aldrich (Madrid, Spain). RCP and 5-OH DCF stock solutions were prepared in DMSO as vehicle, while CPZ and SDS were directly dissolved in ultrapure water (Milli-Q). 6, 12, 24 and 96-well plates were acquired from Labclinics (Barcelona, Spain). Albumin from human serum lyophilized powder, essentially fatty acid free (HSA, CAS no. 70024-90-7), 1,4-diabicyclo-[2.2.2]-octane (DABCO), sodium hydroxide, and Tris(hydroxymethyl)aminomethane were acquired from Sigma-Aldrich (Madrid, Spain). Agarose D1 low EEO was acquired from Condalab (Madrid, Spain). Trichloroacetic acid (TCA) was purchased from Labbox (Barcelona, Spain). EDTA was supplied by Honeywell Fluka (North Carolina, USA) (Valencia, Spain) and 2,4-dinitrophenylhydrazine hydrochloride was provided by Cymit Química S.L. (Barcelona, Spain). 6-carboxy-2′,7′-dichlorodihydrofluorescein diacetate, SYBR Gold DNA and CellMask Orange Plasma stains were supplied by Invitrogen (Madrid, Spain). RedDot Far-red nuclear stain was acquired from Biotium (California, USA). Apo-one Homogeneous Caspase-3/7 assay kit and human immortalized keratinocyte cell line were supplied by ThermoFisher Scientific (Madrid, Spain).

For cell culture experiments, Dulbecco’s Modified Eagle Medium (DMEM, low glucose, phenol red), Dulbecco’s Modified Eagle Medium (DMEM, low glucose, no glutamine, no phenol red), fetal bovine serum (FBS) and penicillin–streptomycin (1 × 10^5^ U/mL, 1 × 10^5^ µg/mL) were supplied by Invitrogen (Madrid, Spain). Trypsin–EDTA (0.25–0.02%) and glutamine (100 µM) solutions were provided by Cultek (Madrid, Spain). Phosphate buffered saline (PBS, pH 7.4, 0.01 M) and neutral red dye were obtained from Sigma-Aldrich. Comet assay lysis solution and flare slides were purchased from R&D systems (Minneapolis, USA).

### Cell culture conditions

Human keratinocytes (HaCaT) were cultured in 75 cm^2^ plastic flasks in DMEM with phenol red supplemented with 10% FBS, 4 mM l-Glutamine and penicillin–streptomycin (100 U/mL, 100 µg/mL) in a humidified incubator (100% relative humidity) at 37 °C under 5% CO_2_ atmosphere. Splitting cells were done twice a week with 1:5 and 2:5 ratios.

### Absorption and emission spectra measurements

Ultraviolet absorption spectra were recorded on a Jasco V-650 UV/Vis spectrophotometer. Measurements were performed in PBS at room temperature using 1 cm quartz cells with 3.5 mL capacity.

Fluorescence in solution and within cells (λ_exc_ = 320 nm) were recorded using a Synergy H1 multi-mode microplate reader in 96-well black plates.

### Irradiation equipment

For all in vitro photosensitization assays, an LCZ-4 photoreactor (Luzchem, Canada), fitted with 14 Hitachi lamps for top and sides (λ_max_ = 350 nm, Gaussian distribution), was used as the UVA light source. Irradiations were carried out using different transparent plates: 96-well (for phototoxicity and caspase-3/7 activation assays), 24-well (for comet assays), 12-well (for ROS assay) and 6-well (for photooxidation assays) plates and they were performed through the lid of the plates, attenuating the direct effect of UVB radiation over the cell cultures. Before performing the experiments, the absorption of the plastic lid of the plates was measured using the microplate reader described above. It was determined that the plastic lid filters all radiation below 310 nm, which contributes to the mitigation of the effect of UVB radiation over the cell cultures. Moreover, to avoid overheating, plates were kept on ice inside the photoreactor.

In photogenotoxicity experiments, cell viability of cultures after irradiation was checked by trypan-blue dyeing to ensure that the results obtained were not false-positive results triggered by DNA fragmentation due to cell death.

### Cellular targeting by confocal microscopy

Human keratinocytes were seeded on glass coverslips in 24-well plates (25,000 cells/well). Next day, cells were incubated in fresh DMEM medium with RCP 20 µM for 60 min, CellMask Orange Plasma membrane stain (dilution 1:10,000) for 30 min and RedDot Far-Red nuclear stain (dilution 1:200) for 10 min. Then, coverslips were washed three times with PBS and mounted in glass slides with a solution of Mowiol, containing 2.5% 1,4-diazobicyclo-[2.2.2]-octane (DABCO). Microscopy and imaging have been done with a Leica SP5 confocal microscope with a sequential mode. The excitation wavelengths for RCP, membrane stain and nuclear stain were 405, 543 and 662 nm, respectively, and emission wavelengths were 480, 567 and 694 nm. Representative images were chosen among two different coverslips from different regions of each slide.

### In vitro phototoxicity assay: neutral red uptake (NRU)

Phototoxicity test was performed according to the OECD guideline 432^19^ using HaCaT cells instead of BALB/c 3T3 fibroblasts cells employed in the standard procedure, owing to the similarity with human skin cells^20^.

Briefly, two 96-well plates were seeded for each compound (20,000 cells/well). The following day, culture media was replaced with the free phenol red DMEM to avoid ultraviolet absorption for itself. Then, cells were treated with test compounds at eight concentrations, ranging from 2.5 to 500 µM, and incubated for 1 h in dark conditions. After that, one plate was irradiated at a 5 J/cm^2^ UVA radiation dose under the conditions described above, whereas the other one was kept in dark conditions. Immediately, all compound solutions were replaced with DMEM medium and plates were incubated overnight. The next day, neutral red solution (50 µg/mL) was added to all wells and incubated for 2 h. Cells were washed once with PBS and neutral red absorbed was extracted from lysosomes in 100 µL with a fresh extraction buffer [Milli-Q water, ethanol and acetic acid 50% (v/v), 49.5% (v/v), 0.5% (v/v), respectively]. Absorbance results were recorded at 550 nm on a Synergy H1 microplate reader and, for all compounds, dose–response curves were made by Boltzmann fitting with GraphPad Prism v.5.03. Thus, it could be determined the concentration of RCP and controls by which the neutral red uptake is reduced to 50% (IC_50_) in dark and UVA light conditions. Finally, the photoirritation factor (PIF) values were calculated using the Eq. (1):

1 $$PIF = \frac{{IC_{50}\, ( {Dark} )}}{{IC_{50}\,( {UVA\, light} )}}$$

Based on the OECD Guideline, a compound is predicted as phototoxic if PIF > 5, probably phototoxic if PIF ≥ 2 and < 5 and non-phototoxic if PIF < 2. As positive and negative controls, CPZ and SDS were used, respectively.

### In vitro reactive oxygen species (ROS) detection upon UVA irradiation

To investigate oxidative stress-induced phototoxicity, ROS activation assay was performed using a pro-fluorescent substrate (carboxy-H_2_DCF-DA), which is deacetylated (carboxy-H_2_DCF) by cellular esterases and then, it is subsequently oxidated by ROS to 6-carboxy-2′,7′-dichlorofluorescein (carboxy-DCF), a fluorescent compound unable to run away from the intracellular matrix.

For this experiment, HaCaT cells were seeded in two 12-well plates (40,000 cells/well). Next day, cells were incubated in fresh free phenol red DMEM with increasing amounts of RCP (2, 5 and 10 µM) for 1 h in dark conditions. After that, one of them was irradiated at a 5 J/cm^2^ UVA light dose under the conditions described above, keeping the other one in dark conditions. Afterward, media was replaced by a solution of 6-carboxy-2′,7′-dichlorodihydrofluorescein diacetate (carboxy-H_2_DCF-DA, 25 µM) in PBS and incubated for 30 min (λ_exc_ = 495 nm, λ_em_ = 525 nm). Finally, all wells were washed twice in PBS and fluorescence images were acquired with a Leica DMI 4000B fluorescence microscopy using the Fluorescein FITC filter. Representative images were chosen among two different wells from different regions of each condition.

### Protein carbonyl content test

Detection of carbonylated proteins was carried out following the protein carbonyl derivatization assay through 2,4-dinitrophenylhydrazine (DNPH)^21^, with minor modifications. Briefly, solutions of HSA (1 mg) in PBS (200 μL) were prepared in the absence or in the presence of 5 or 10 μM of RCP and irradiated with a UVA dose of 5, 10 and 15 J/cm^2^, or in dark conditions. Immediately, 200 μL of DNPH 10 mM was added to the samples, mixed with vortex and kept in darkness at room temperature for 60 min. Then, 1 mL of TCA (20% v/v) was added to protein samples, incubated on ice for 15 min, vortex mixed and centrifugated at 13,000 rpm for 5 min. Next, supernatants were discarded and pellets were washed twice with 1 mL of ethanol/ethyl acetate 50:50 (v/v), containing TCA 20%, mixed by vortex and centrifugated again in order to remove any free DNPH. Protein pellets were dried in a heater at 60 °C for 15 min to allow complete solvent evaporation and resuspended in 100 μL of 6 M guanidine hydrochloride. Once protein pellets were completely dissolved (stayed overnight at 4 °C), absorbance at 375 nm was recorded using the Synergy H1 microplate reader and carbonyl contents were expressed as nmol of carbonyl per mg protein according to Eq. (2):

2 $$Carbonyl \, content \left( {{\raise0.7ex\hbox{${nmol}$} \!\mathord{\left/ {\vphantom {{nmol} {mg\, protein}}}\right.\kern-\nulldelimiterspace} \!\lower0.7ex\hbox{${mg\, protein}$}}} \right) = \frac{{( {A_{sample} - A_{blank} } ) \cdot 100}}{6.364}$$

where 6.364 is the ε at 375 nm × l (the length of the path light for a 96-well plate).

### Assessment of nuclear DNA damage by comet assay

The single cell gel electrophoresis assay, also known as comet assay, was performed following the methodology previously described^18^ with slight modifications. Cells were trypsinized, resuspended in PBS and placed on ice for 2 h to allow HaCaT cells to repair mild DNA damage induced by trypsin. Then, cells (50,000 cells/well in two 24-well plates) were seeded and treated with RCP (2, 5, 10, 50 μM) or chlorpromazine (CPZ, 4, 10 μM), which it was used as a positive control. After 30 min of the incubation at 4 °C in darkness, one plate was placed in the photoreactor in order to irradiate the cells on ice (2 or 5 J/cm^2^), whereas the other one was kept in the absence of light as negative control. Next, 100 μL of each cell suspension were mixed carefully with 100 μL of 1% low melting point agarose solution and drops were loaded onto Trevigen treated slides and placed on ice-cold tray to allow its jellification. In parallel, cell viability after irradiation was checked by trypan blue exclusion assay^22^. Then, the slides were immersed in coupling jars containing cold lysis buffer (2.5 M NaCl, 0.1 M Na_2_EDTA, 0.01 M Tris, 1% Triton X-100 in distilled water and pH 10) and overnight incubated at 4 °C to promote cell lysis. Next day, all slides were placed in a Trevigen comet assay electrophoresis tank, covered with 1 L of cold alkaline electrophoresis buffer (0.2 M NaOH, 1 mM EDTA in distilled water reaching a pH ≥ 13). The electrophoresis was run at 21 V (≈ 300 mA) for 30 min at 4 °C. When the electrophoresis finished, the slides were washed twice with Milli-Q water for 5 min; DNA was fixed by slide incubation in 70% ethanol for 5 min followed by other 5 min in 100% ethanol and dried in a heater at 37 °C for 2 h. Finally, comet nucleoids and tails were stained by incubating the slides in a SYBR Gold (1:10,000 TE buffer) bath for 30 min, washed once with Milli-Q water, air dried, and kept in darkness until further visualization. Leica DMI 4000B fluorescence microscope was used for nucleoids and tails DNA visualization, and at least five pictures were taken for each sample. Finally, DNA % in tail as a measure of DNA damage was calculated for each condition with the analysis of at least 100 DNA comets by visual scoring, utilizing the open-source counter software ImageJ v1.52p. Total comet score (TCS) was determined with the classification of six DNA damage categories^23^ with the following Eq. (3):

3 $$TCS = \frac{{\mathop \sum \nolimits_{n = 1}^{6} class \, n \, comet \times n}}{6}$$

The final results were denoted in 1–100 arbitrary units, where class 0 comets show no DNA damage and class 6 comets indicate the maximum DNA damage.

Additionally, to determine if cell culture were able to repair the DNA damage promoted by compounds + UVA light, 5-OH DCF (100 µM) was used as recovery positive control^16,17^. Thus, before the cellular lysis, slides were embedded with DMEM, freed of supplements, and incubated at 37 °C for 6 h. Then, slides were immersed in the same lysis buffer for at least 2 h to allow cellular lysis and the same procedure was followed as describe above.

### Caspase-3/7 activation assay

In brief, two 96-well plates were seeded (20,000 cells/well). Next day, culture media was replaced with free phenol red DMEM to avoid absorption for itself. Then, cells were treated with RCP at 0.25, 1 or 3 μM and incubated for 1 h in dark conditions. After that, one plate was irradiated at a 5 J/cm^2^ UVA radiation dose, whereas the other one was kept in dark conditions. Immediately, all solutions were replaced with DMEM medium and plates were incubated overnight. The following day, 100 μL of caspase-3/7 substrate (bis-N-CBZ-l-aspartyl-l-glutamyl-l-valyl-l-aspartic acid amide; Z-DEVD-R110), diluted in Apo-one Homogeneous Caspase-3/7 Buffer (R&D Systems) was added to each well and incubated for 4 h. Then, fluorescence of rhodamine 110 released by caspase activity was monitored using a Synergy H1 microplate reader in 96-well black plates (λ_exc_ = 485 nm, λ_em_ = 528 nm).

### Data analysis and statistics

Results obtained are expressed as mean ± standard deviation from at least four independent experiments. Data were analyzed and regression methods were developed using GraphPad Prism version 5.03 for Windows (https://www.graphpad.com). Statistical significance was calculated by the Student’s *t* test, considering only *p* values lower than 0.05 as significant results (*p < 0.05; **p < 0.01; ***p < 0.001). Images were analyzed using ImageJ version 1.52p for Windows (https://imagej.nih.gov/ij/).

## Results and discussion

### Cellular internalization of RCP

**Figure 2:**
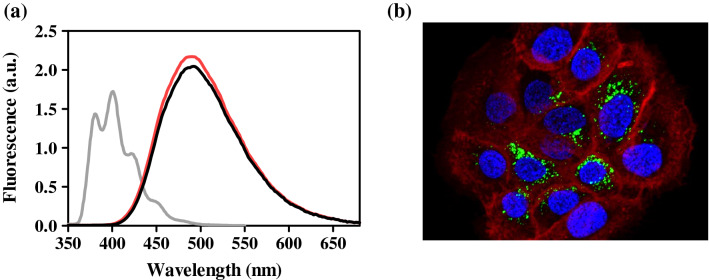
(**a**) Emission spectra (λ_exc_ = 320 nm) of rucaparib (RCP) after internalization on HaCaT cells (red), RCP in PBS solution (black) and anthracene (grey) in ethanol. The latter was used as standard. Isoabsorptive solutions for all conditions were prepared and then, fluorescence was recorded. (**b**) Intracellular localization of RCP in HaCaT cells by confocal microscopy. Keratinocytes, seeded on glass coverslips, were incubated with 20 µM solutions of RCP (green) and labelled with CellMask Orange Plasma membrane (red) and RedDot1 Far-Red nuclear (blue). Colours were chosen arbitrarily. Emission spectra were drawn using GraphPad Prism v5.03.

Firstly, emission spectrum of RCP was recorded in both PBS and cells to check that its intrinsic fluorescence could be used for confocal microscopy experiments. For this purpose, HaCaT culture was treated with RCP to ensure both the uptake by cells and fluorescence properties after internalization. Emission spectra (λ_exc_ = 320 nm) of RCP was recorded in PBS solution and after internalization into HaCaT cells and the fluorescence quantum yield (*ϕ*_F_) was determined in presence and absence of cells (Fig. 2a) by comparison with anthracene in ethanol as standard^24^. Hence, RCP showed an intracellular fluorescence with a maximum emission around 480 nm and *ϕ*_F_ = 0.696 ± 0.018, which is similar to that observed in aqueous solution (*ϕ*_F_ = 0.651 ± 0.021). As expected for the chemical structure of RCP (heterocyclic amide, fluorine and aromatic ring linked to core indole), its fluorescence spectrum undergoes a substantial redshift in comparison with the indole moiety that is similar to tryptophan^25^.

Hence, confocal microscopy was employed in order to analyze the intracellular localization of RCP using it intrinsic fluorescence properties and CellMask Orange Plasma and RedDot Far-Red to stain membrane and nucleus, respectively. In this context, 405, 543 and 662 nm were used as excitation wavelengths for RCP, membrane stain and nuclear stain, respectively. As emission wavelengths, 480, 567 and 694 nm were selected for RCP, membrane stain and nuclear stain, respectively. After 1 h of incubation, an efficient uptake by HaCaT cells was observed, showing a perinuclear distribution as it was illustrated in Fig. 2b.

### Cellular phototoxicity assessment of RCP

#### In vitro neutral red uptake, NRU

A phototoxicity test was performed to evaluate the phototoxic potential of RCP in combination with UV radiation. For this purpose, cell viability of HaCaT cells treated with increasing concentrations of RCP was measured, using neutral red as a vital dye, under dark or UVA light conditions. Therefore, the half-maximal inhibitory concentrations (IC_50_) were determined from dose–response curves (Fig. S1). Finally, the photoirritation factor (PIF), which corresponds to the ratio of the IC_50_ under dark or light conditions, was calculated using the Eq. (1).

**Figure 3:**
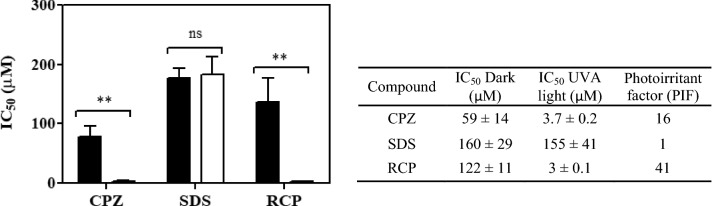
In vitro NRU phototoxicity assay of rucaparib (RCP). IC_50_ values were established from dose–response curves and PIF values from the ratio of IC_50_ under dark or light conditions. For positive and negative controls, chlorpromazine (CPZ) and sodium dodecyl sulphate (SDS) compounds were used, respectively. Data represent mean ± SD of four independent experiments performed in triplicate upon dark (■) or UVA light (□) conditions. Asterisks indicate significant differences by Student’s *t* test (*ns* non-significant, **p < 0.01). Data were analyzed using GraphPad Prism v5.03. According to the OECD 432 guideline (2019), PIF < 2 means “no phototoxicity”, 2 < PIF < 5 means “probable phototoxicity” and PIF > 5 means “phototoxicity”.

The results obtained are shown in Fig. 3, where RCP was shown to be clearly phototoxic (IC_50_ Dark = 122 ± 11 µM, IC_50_ UVA light = 3 ± 0.1 µM), with a PIF value of *ca.* 41, three times higher than chlorpromazine (CPZ) value, which was used as a positive control. Although RCP was significantly less damaging than CPZ within dark conditions, both drugs exhibit similar IC_50_ (3 and 3.7 respectively) after UVA irradiation, in agreement with the highest RCP PIF value.

Induced drug phototoxicity can provoke damage to cells, especially to key cellular components including lipid, protein, and DNA. Bearing in mind that lipid photoperoxidation was not observed using the thiobarbituric acid (TBA) method^26,27^ (Fig. S2); probably the higher phototoxicity observed could be attributed to both proteins and genomic DNA.

#### In vitro ROS generation by rucaparib

Taking into consideration that RCP displays toxicity under sunlight exposure, it seems interesting to explore the possibility of reactive oxygen species (ROS) generation after UVA radiation, which can be responsible for the oxidation of biological molecules and cell membranes.

**Figure 4:**
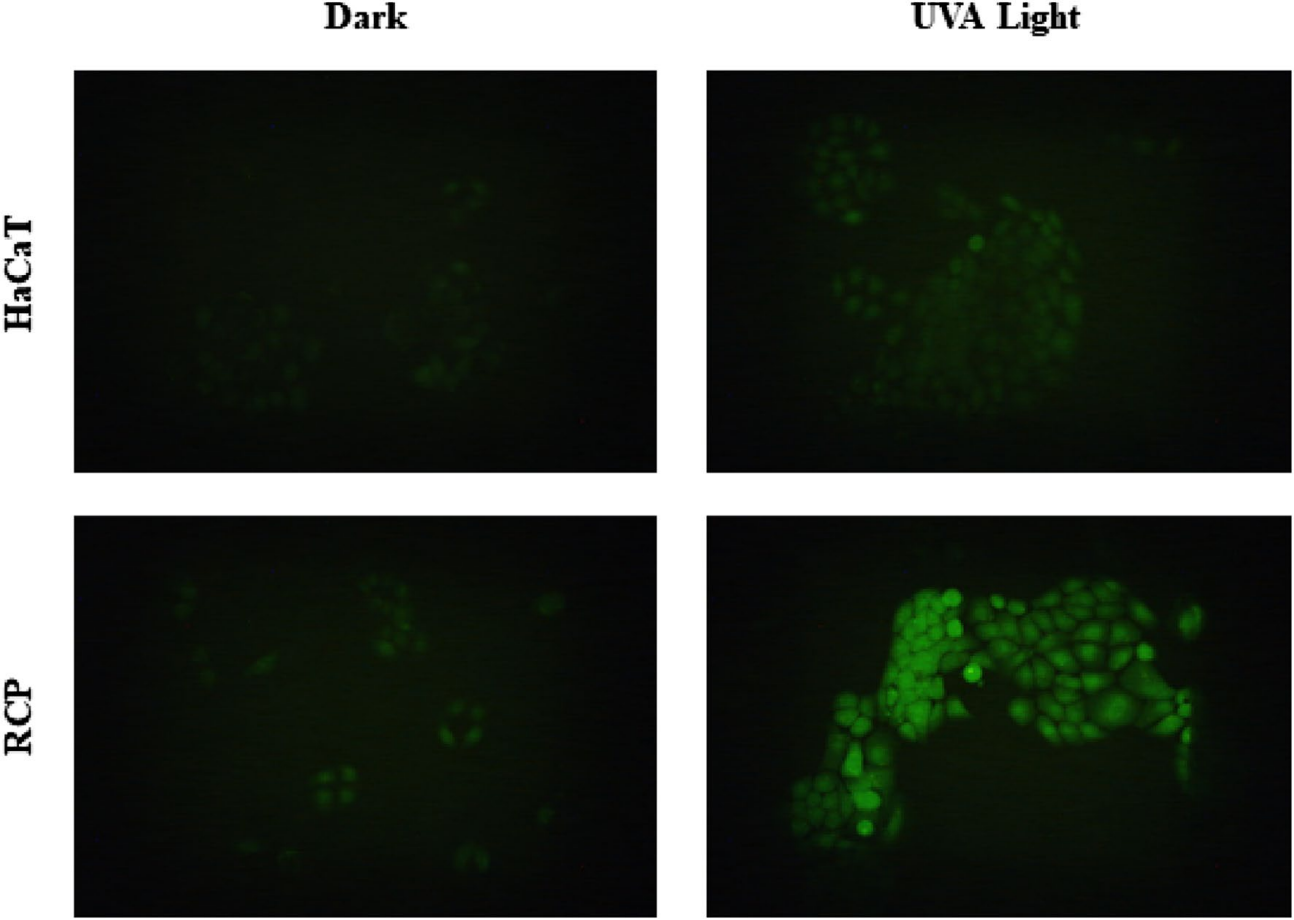
Representative fluorescence microscopy images (Fluorescein FITC filter) of ROS experiments. Human keratinocytes (HaCaT) were seeded on 12-well plates and incubated in the presence (10 µM) or absence of rucaparib (RCP); HaCaT cells were treated with 25 µM of 6-carboxy-2′,7′-dichlodihydrorofluorescein diacetate (carboxy-H_2_DCF-DA). Dark: Non-irradiated cells. UVA Light: cells irradiated with UVA Light (5 J/cm^2^).

To this purpose, cells were staining with carboxy-H_2_DCF-DA after incubation with different concentrations of RCP and irradiated with a UVA light dose of 5 J/cm^2^. Finally, to analyze ROS activity, the fluorescence of carboxy-DCF was recorded by means of fluorescence microscopy using a Fluorescein FITC filter. Images displayed on the left in Fig. 4 illustrate that ROS noticed in dark conditions are negligible in both the absence or presence of RCP. This fact is as expected since the IC_50_ for RCP under dark conditions was found to be 122 µM. Moreover, when cells are irradiated with a UVA dose of 5 J/cm^2^, the lack of fluorescence signal in non-treated cells indicates the suitability of the experiment (photograph on the right and up). In contrast, after cells treatment with RCP (10 µM) followed by irradiation, a broad fluorescence signal was registered, which exhibits the capability of RCP to induce ROS after UVA radiation (photograph on the right and bottom).

To a deeper investigation, another set of experiments was conducted using 2, 5 and 10 µM as concentrations of RCP. Results are available in the supporting section (see Fig. S3), where it can be appreciated an increase of ROS production as the drug concentration increases as well.

#### Protein photooxidation

Protein carbonylation represents the most frequent and usually irreversible oxidative modification affecting proteins. It is known that RCP binds to plasma protein, and therefore it makes sense to determine protein photooxidation by the measurement of the carbonyl content, as an early biomarker of oxidative damage, using the 2,4-dinitrophenylhydrazine (DNPH) derivatization method. In this context, PBS solutions containing HSA (0.075 μM) and RCP (5 or 10 μM) were irradiated with different UVA light doses (5, 10 and 15 J/cm^2^) and subsequently carbonyl content was measured. For each RCP concentration, carbonyl concentration generated in situ was fitted to a zero-order kinetic: the levels of carbonyl moiety were directly proportional to the amount of UVA dose. The irradiation dose has been calculated by the Eq. (4), where the irradiance can be established multiplying the reading of a power meter detector by the calibration factor.

4 $$Irradiation\, dose \left( {\frac{J}{{cm^{2} }}} \right) = \frac{{Irradiation\, time ( {min}) \cdot Irradiance \left( {\frac{mW}{{cm^{2} }}} \right) \cdot 60}}{1000}$$

Rate constant values (k) for RCP at 5 µM and 10 µM were found to be 0.033 and 0.056 nmol·min/mg protein, respectively.

Moreover, taking into account that HSA absorption in the UVA region is negligible, both dark and irradiated conditions contained similar levels of carbonyl moiety; this indicates the suitability of the UVA dose employed to prevent false-positive results.

**Figure 5:**
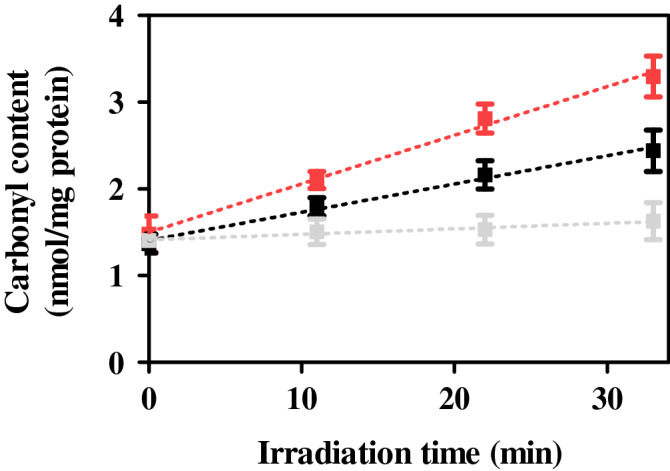
Protein photooxidation by rucaparib (RCP) determined by the measurement of the carbonyl content using the DNPH derivatization method. HSA solutions (0.075 μM) in the absence (grey) or in the presence of RCP at 5 μM (black) or 10 μM (red) were irradiated at different irradiation times. Linear regressions were established using GraphPad Prism v5.03.

As shown in Fig. 5 and Fig. S4, RCP increased significantly the carbonyl concentration in HSA after UVA irradiation (up to threefold), clearly pointing out the capability of this drug to promote photooxidation in cellular membranes. These results are in agreement with those obtained by the NRU test, in which RCP exhibited substantial phototoxicity.

#### Photogenotoxicity of RCP

Comet assay under alkaline conditions is a well-known technique employed to reveal single-strand breaks (SSB), double-strand breaks (DSB) and alkali-labile sites on chromosomic DNA of an individual cell^28^. Thus, for detecting DNA photodamage in single cells, human keratinocytes were incubated for 30 min with RCP. Then, they were exposed for 5 min at UVA irradiation (2 J/cm^2^) and embedded in agarose on a slide. To avoid misleading results as cell death also promotes DNA fragmentation by activation of caspase-activated DNases (CADs), cell viability after treatment plus irradiation was routinely assessed by trypan blue exclusion assay^22^, giving a viability rate higher than 85%, which indicates the suitability of the UV dose and drug concentration chosen. Thus, this is in agreement with that reported from the literature, where it is indicated that the cell viability has to be at least 70–80% to ensure reliable comet assay results^29,30^.

Next, alkaline electrophoresis was carried out to allow the damaged and fragmented DNA to migrate away from the nucleus. Upon staining with SYBR Gold, the fluorescence of the comet nucleoids and tails were analyzed by a fluorescence microscope using the Fluorescin FITC filter. Percentage of DNA damage was calculated using the visual scoring (six DNA damage categories)^23^ of at least 100 DNA comets.

**Figure 6:**
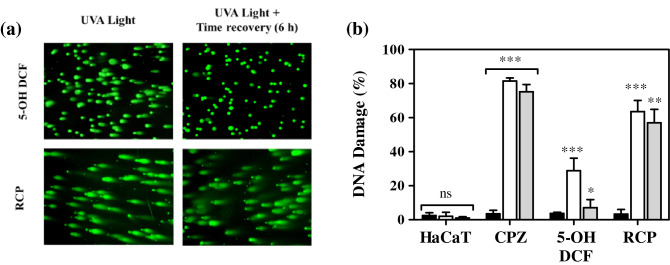
Unexposed HaCaT cells alone or treated with 10 μM of chlorpromazine (CPZ, positive control), 100 μM of 5-hydroxydiclofenac (5-OH DCF, recovery positive control) or 50 μM of rucaparib (RCP). Cells were kept on dark conditions or irradiated with a 2 J/cm^2^ UVA dose. (**a**) Representative fluorescence microscopy images of cells treated with RCP or 5-OH DCF. (**b**) Percentage of DNA damage by visual scoring of untreated cells (HaCaT) or treated with CPZ, 5-OH DCF or RCP. Data represent mean ± SD of four independent experiments upon dark (■), UVA light (□) or UVA light + 6 h of time recovery  conditions. Asterisks indicate significant differences by Student’s *t* test (***p < 0.001, **p < 0.001, *p < 0.05, *ns* non-significant). Visual scoring of DNA damage was established using ImageJ v1.52p. The percentage of DNA damage was represented using GraphPad Prism v5.03.

As displayed in Fig. 6 and Fig. S5, comet assay revealed significant damage promoted by RCP in combination with UVA light (around 60%). To combat DNA damage, cells have developed several DNA-repair pathways; however, if the repair is defective DNA lesions may lead to mutations. Therefore, to assess whether the HaCaT cells can repair photoinduced damage to DNA by RCP, an additional set of comet assays were carried out after irradiation followed by 6 h cell recovery. It is remarkable that after 6 h of time recovery, no substantial reduction in nuclear DNA damage was noticed, indicating non-reversible DNA damage.

Furthermore, to compare the photosensitized DNA damage generated using lower concentrations of RCP close to its IC_50_ (2, 5, and 10 μM), a new set of experiments was carried out using the same UVA dose that the employed for cell viability NRU assay. Despite the conditions were different, as it can be observed comparing both experiments (50 µM at 2 J/cm^2^ and 5–10 µM at 5 J/cm^2^), a good correlation was obtained, determining in both cases a percentage of DNA damage around 60%. More details are available in the supporting information (Fig. S6).

#### Cellular apoptosis induced by RCP

**Figure 7:**
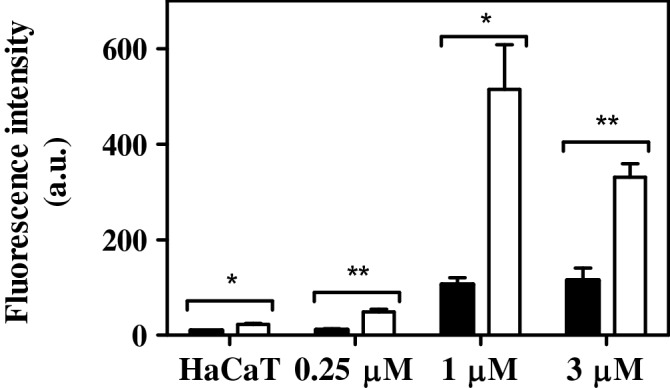
Relative fluorescence of rhodamine 110 (λ_exc_ = 485 nm, λ_em_ = 528 nm) after caspase-3/7 activation assay by cells treated with rucaparib (RCP). Cells unexposed (HaCaT) or treated with 0.25, 1 or 3 μM of RCP were kept on dark conditions (■) or irradiated at a 5 J/cm^2^ UVA dose (□) and subsequently incubated with Z-DEVD-R110 for 4 h. Data represent mean ± SD of two independent experiments performed in triplicate upon dark or UVA light conditions. Asterisks indicate significant differences by Student’s *t* test (**p < 0.001, *p < 0.05). Data were analyzed using GraphPad Prism v5.03.

Caspase-3/7 activation assay was performed to investigate the molecular mechanism of cell death^31^ induced by RCP in combination with UVA radiation. Thus, apoptosis is a well-known mechanism of “programmed cell death”, where caspase-3/7 activities are responsible for this event and their overexpression conducts irreversibly to the cellular death. In this context, the activity of caspase-3/7 was quantified by a pro-fluorescent substrate (Z-DEVD-R110) that in the presence of caspase enzymes releases a highly fluorescent compound, rhodamine 110 (R110). Hence, cellular apoptosis was determined indirectly by the measurement of the R110 fluorescence intensity after its releasement by cells treated with different concentrations of RCP. As shown in Fig. 7, an increase of caspase-3/7 activity was observed after cell treatment with RCP and subsequently UVA radiation. It is interesting to note that the caspase-3/7 maximal activity is observed at 1 μM of RCP, which corresponds to a concentration lesser than its UVA IC_50_ (3 μM). However, when this concentration is closed to its IC_50_, the enzyme activity is reduced due to the phototoxicity of the drug that provokes cell death indeed.

## Conclusion

In summary, the present work has demonstrated that the anticancer drug RCP has the ability to trigger photosensitivity reactions. On one hand, the in vitro NRU cell viability test has proven the high phototoxic potential of RCP. This phototoxicity can be attributed to photosensitized damage towards main cellular biomolecules (lipids, proteins and DNA). Accordingly, lipid photo peroxidation assessed by the thiobarbituric acid method is negligible. Moreover, RCP photosensitized oxidation of HSA is established using the protein carbonylation method, and the photodamage to DNA induced by RCP is revealed using the comet assay. Thus, RCP results to be both phototoxic and photogenotoxic.

On the other hand, RCP is able to generate reactive oxygen species after UVA radiation, which could lead to stress-oxidative damage to subsequent cell death. In this context, the apoptosis cell death mechanism induced by combining RCP and UVA radiation is noted by experiments with caspases.

It is worth noting that the results are highly relevant concerning RCP photosafety and they result clinically interesting to identify the cutaneous adverse events associated with targeted therapies. Besides, this is the first example of a PARP inhibitor able to induce photosensitized damage to biomolecules. The employed methodology can be extended to other PARP inhibitors in order to assess possible photo(geno)toxicity reactions.

## Supplementary Information

Supplementary Figures.
