# Supplementary material for: Evaluation of phototoxicity induced by the anticancer drug rucaparib
Source: Sci Rep. 2022 Mar 2;12:3434. doi: 10.1038/s41598-022-07319-9 (PMC8891269; doi:10.1038/s41598-022-07319-9)
Supplement: Supplementary file 1 — Supplementary Figures. [file 41598_2022_7319_MOESM1_ESM.pdf]

SUPPLEMENTARY MATERIAL

Evaluation of phototoxicity induced by the anticancer drug rucaparib

Alejandro Mateos-Pujante,[1,2] María Consuelo Jiménez,[1,2,*] and Inmaculada Andreu[1,2,*]

[1]Departamento de Química-Universitat Politècnica de València, Camino de Vera s/n, 46022, Valencia, Spain

[2]Unidad Mixta de Investigación Universitat Politècnica de València -Instituto de Investigación Sanitaria (IIS) La Fe, Hospital Universitari i Politècnic La Fe, Avenida de Fernando Abril Martorell 106, 46026, Valencia, Spain

*****Correspondence should be addressed to M. C. J. (mcjimene@qim.upv.es) or I. A. (iandreur@qim.upv.es).

ORCID A. Mateos-Pujante: 0000-0003-1571-7162

ORCID M .C. Jiménez: 0000-0002-8057-4316

ORCID I. Andreu: 0000-0003-3409-9443

Table of contents:

Fig. S1 Dose-response curves for cell viability of rucaparib.

Fig. S2 Lipid photooxidation by rucaparib.

Fig. S3 Fluorescence microscopy images of ROS experiments.

Fig. S4 Protein photooxidation by rucaparib.

Fig. S5 Fluorescence microscopy images of comet assay experiments.

Fig. S6 Comet assay performed with an UVA dose of 5 J/cm^2.

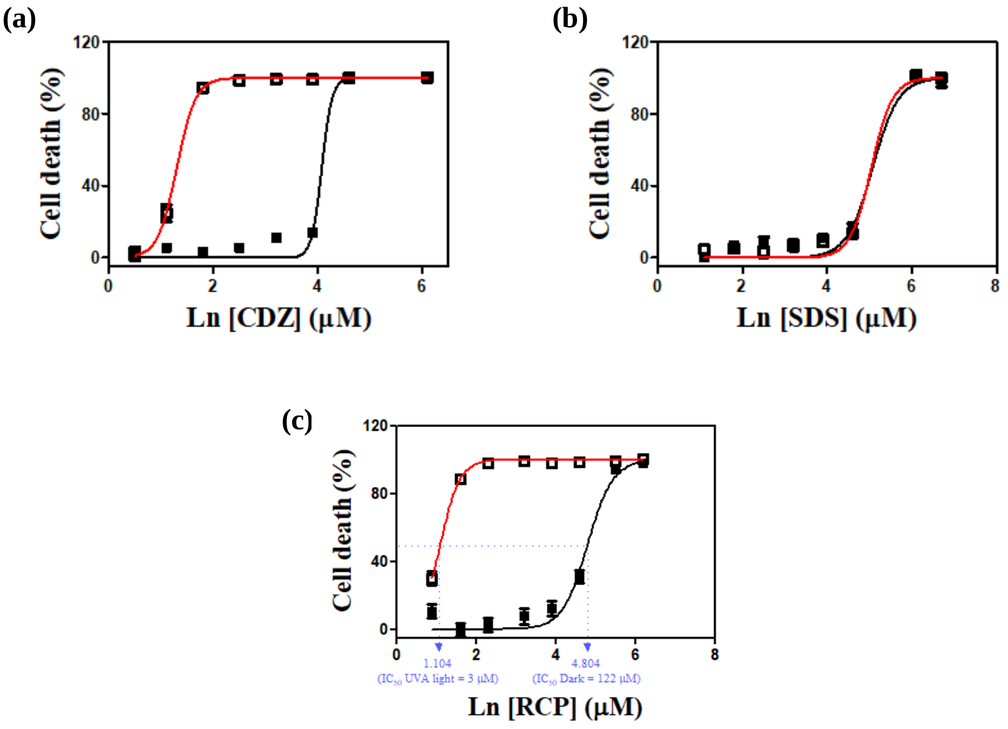

Fig.S1 Cell viability dose-response curves of HaCaT cells treated with rucaparib (RCP, **c**). Chlorpromazine (CPZ, **a**) and sodium dodecyl sulfate (SDS, **b**) were used as positive and negative controls of phototoxicity in the presence (□) or absence (■) of UVA Light (5 J/cm^2). Data represent the mean ± SD from four independent experiments performed in triplicate.

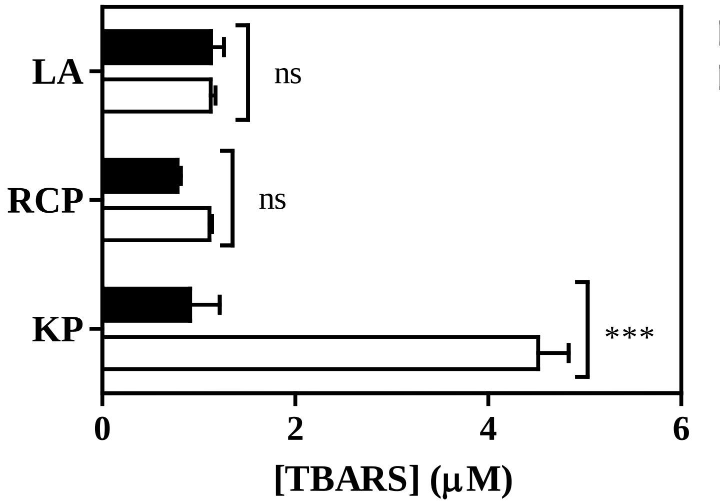

Fig. S2 Lipid photoperoxidation by rucaparib (RCP). Linoleic acid (LA) solutions (1 mM) alone or in the presence of RCP (50 µM) were irradiated at a 15 J/cm^2 UVA dose. For positive control, ketoprofen (KP, 100 µM) was used as a positive control. The lipid photoperoxidation was determined by the measurement of the 2-thiobarbituric acid reactive substance (TBARS) using the TBA method. Data represent mean ± SD of two independent experiments performed in duplicate upon dark (■) or UVA light (□) conditions. Asterisks indicate significant differences by Student's t-test (ns: non-significant, ***p < 0.001)

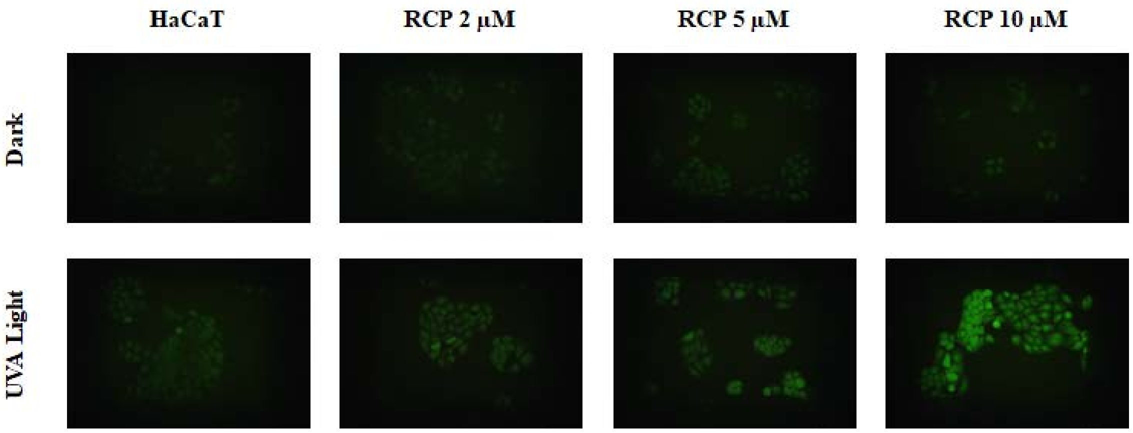

Fig.S3 Representative fluorescence microscopy images (Fluorescein FITC filter) of ROS experiments. Human keratinocytes (HaCaT) were seeded on 12-well plates and incubated in the presence (2, 5 or 10 µM) or absence of rucaparib (RCP); HaCaT cells were treated with 25 µM of 6-carboxy-2’,7’-dichlorodihydrofluorescein diacetate (carboxy-H_2DCF-DA). Dark: Non-irradiated cells. UVA Light: Cells irradiated with UVA Light (5 J/cm^2).

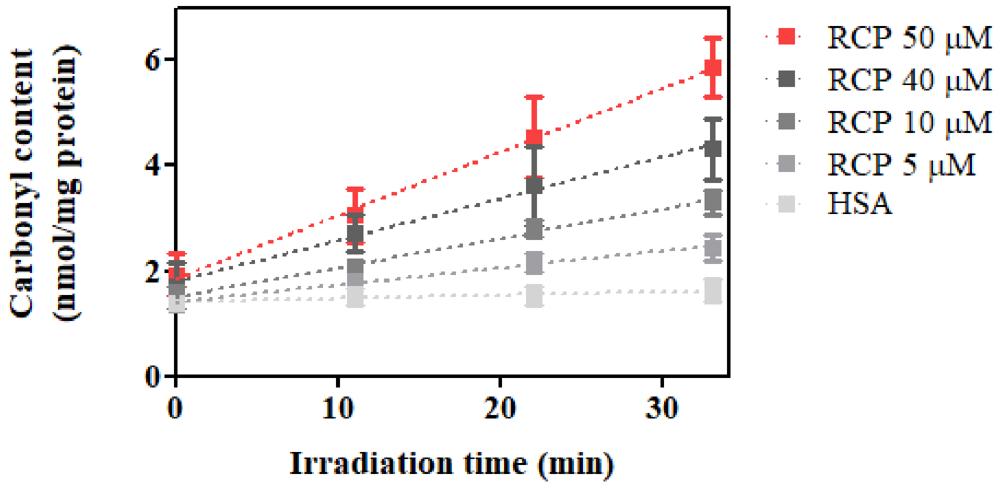

Fig. S4 Protein photooxidation by rucaparib (RCP). HSA solutions (0.075 µM) alone or in the presence of RCP (5, 10, 40 or 50 µM) were irradiated at 11, 22 and 33 minutes, corresponding to a 5, 10 and 15 J/cm^2 UVA dose, respectively. The protein photooxidation was established by the measurement of the carbonyl content using the 2,4-dinitrophenylhydrazine (DNPH) derivatization method. Data represent mean ± SD of three independent experiments performed in triplicate.

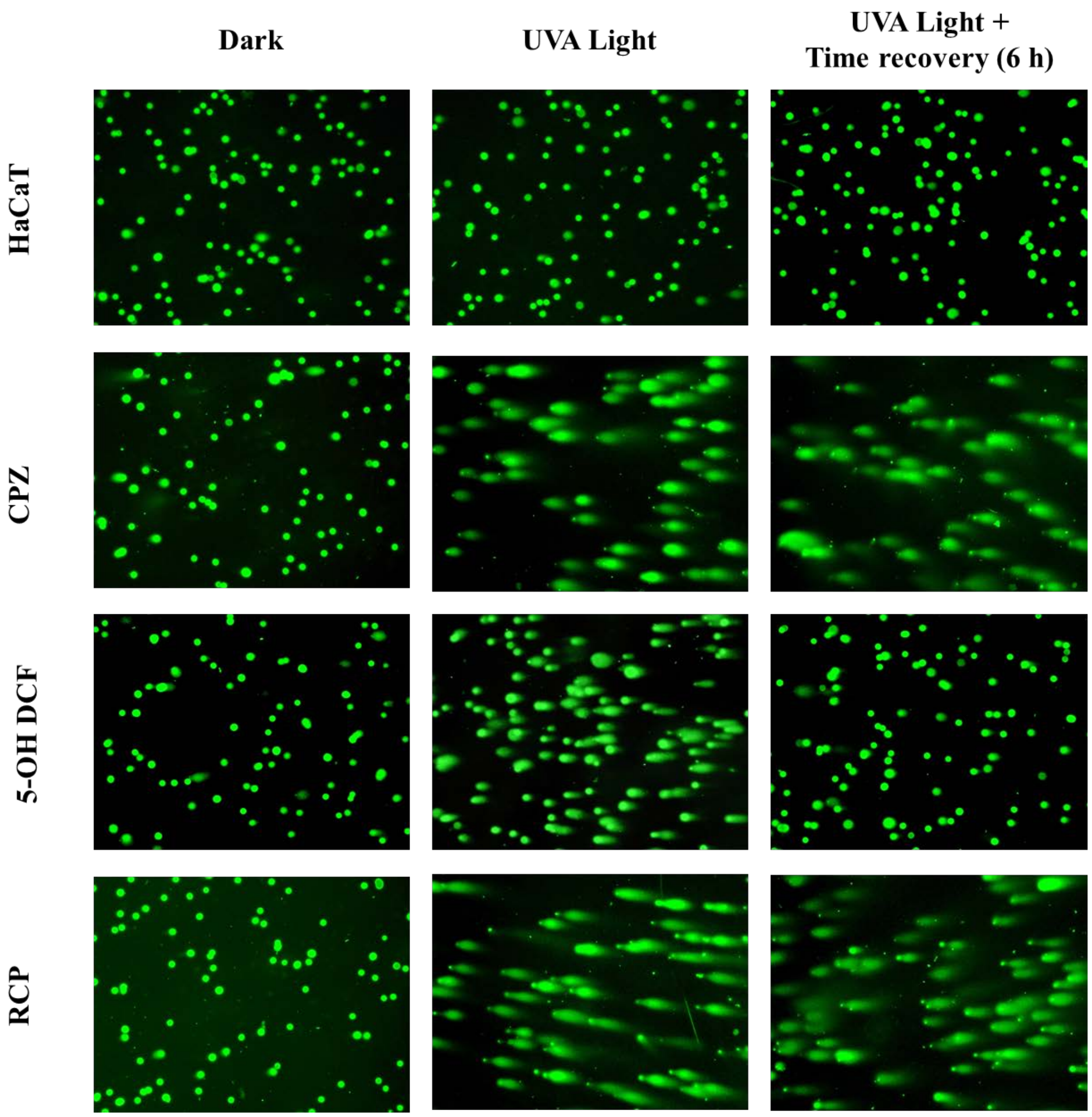

Fig. S5 Representative fluorescence microscopy images (fluorescein FITC filter) of comet assay experiments. HaCaT: Human keratinocytes without treatment. CPZ: Cells treated with chlorpromazine (10 µM). 5-OH DCF: Cells treated with 5-hydroxydiclofenac (100 µM). RCP: Cells treated with RCP (50 µM). Dark: Non-irradiated Cells. UVA Light: Cells irradiated with UVA Light (2 J/cm^2). UVA Light + Time recovery (6 h): Cells irradiated with UVA Light (2 J/cm^2) followed by a 6 h-incubation at 37 °C.

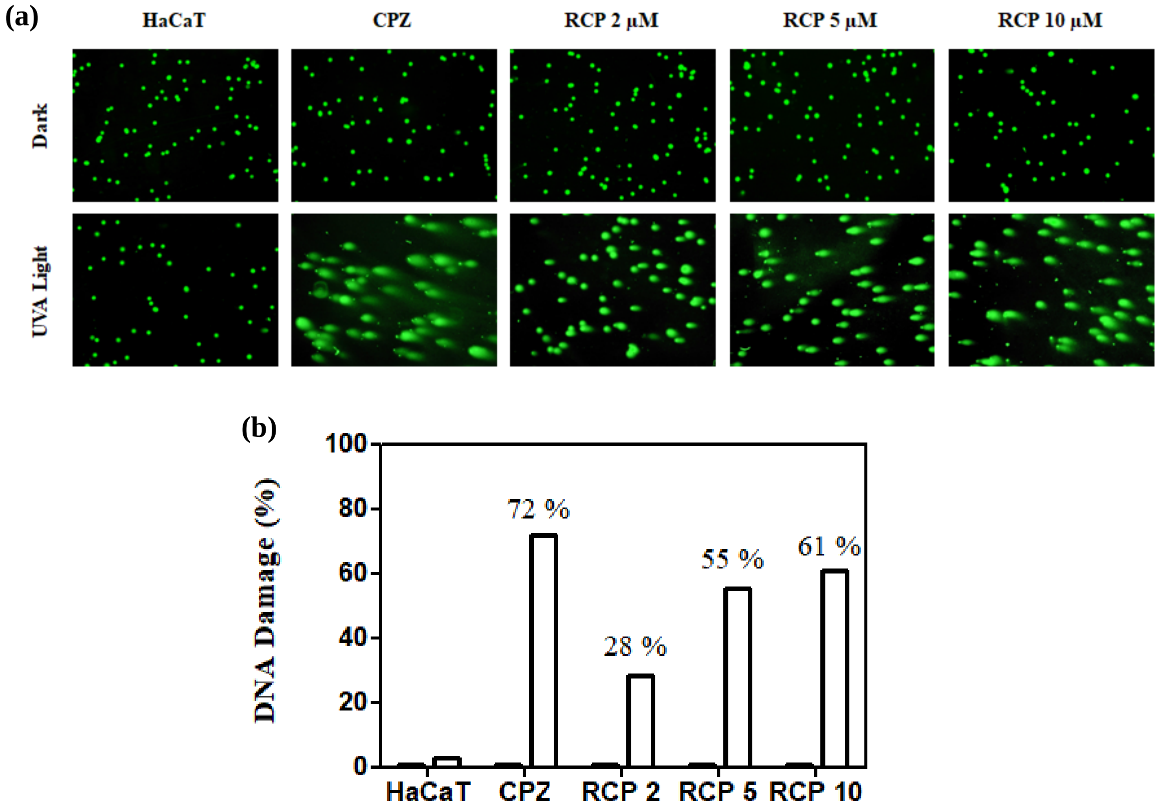

Fig. S6 Comet assay performed with an UVA dose of 5 J/cm^2. **a** Representative fluorescence microscopy images (fluorescein FITC filter) of comet assay experiments. HaCaT: Human keratinocytes without treatment. CPZ: Cells treated with chlorpromazine (5 µM). RCP: Cells treated with RCP (2, 5 or 10 µM). Dark: Non-irradiated Cells. UVA Light: Cells irradiated with UVA Light (5 J/cm^2). **b** Percentage of DNA damage by visual scoring of untreated cells (HaCaT) or treated with CPZ or RCP upon dark (■) or UVA light (□).
